# Effects of summer supplementation of *Houttuynia cordata* extract on growth performance, anti-inflammatory properties, and rumen fermentation in Guizhou black goats

**DOI:** 10.3389/fvets.2025.1627331

**Published:** 2025-07-03

**Authors:** Lingling Xie, Zhongling Jian, Mingyan Tang, Chen Li, Zhiyu Huang, Fu Wang, Ren Ou, Shengyong Lu

**Affiliations:** ^1^Guizhou Provincial Breeding Livestock and Poultry Germplasm Determination Center, Guiyang, China; ^2^Guizhou Weierlai Testing Technology Co., Ltd., Guiyang, China; ^3^Guizhou Provincial Livestock and Poultry Genetic Resources Management Station, Guiyang, China

**Keywords:** antioxidant, goat, growth performance, heat stress, *Houttuynia cordata* extract

## Abstract

The aim of this study was to examine the effects of supplementing Guizhou black goats with *Houttuynia cordata* extract (HCE) during summer on growth performance, anti-inflammatory activity, and rumen fermentation parameters. A completely randomized single-factor experimental design was employed. Twenty-four healthy Guizhou black goats, with similar body weights (16.03 ± 0.79 kg), were randomly divided into three groups, with eight replicates per group and one goat per replicate. The control group (CON) was fed a basal diet, the LC and HC groups received the basal diet supplemented with 500 mg/kg and 1,000 mg/kg of HCE, respectively. The ADG in the HC group was significantly higher (*p* < 0.05) than that in the CON group. The digestibility of DM in both the LC and HC groups was significantly higher (*p* < 0.05) than in the CON group. Additionally, the digestibility of CP, GE, and NDF in the HC group was significantly higher (*p* < 0.05) than in the CON group. GSH-Px levels in both the LC and HC groups were significantly higher (*p* < 0.05) than in the CON group. T-AOC in the HC group was also significantly higher (*p* < 0.05) than in the CON group. MDA levels in the HC group were significantly lower (*p* < 0.05) than in both the LC and CON groups (*p* < 0.05). The TP content in the HC group was significantly higher (*p* < 0.05) than in the CON group. IgA levels in both the LC and HC groups were significantly higher (*p* < 0.05) than in the CON group. The levels of IL-6 and IL-8 in the HC group were significantly lower (*p* < 0.05) than in the LC and CON groups. Interestingly, the IL-10 level in the HC group was significantly higher (*p* < 0.05) than in the LC and CON groups. The TNF-*α* level in the HC group was significantly lower (*p* < 0.05) than in the CON group. The HCE had no significant (*p* > 0.05) effect on rumen pH, NH_3_-N, and VFAs. In conclusion, a high dose of HCE improved growth performance, apparent nutrient digestibility, and enhanced antioxidant, immune, and anti-inflammatory responses in goats.

## 1 Introduction

Guizhou generally has a humid subtropical temperate climate, with summer maximum temperatures reaching up to 32.75°C^1^ such high temperatures can induce heat stress in animals (1). Heat stress negatively affects feed intake, antioxidant systems, mitochondrial function, and heat shock protein expression. It disrupts the body’s redox balance and reorganizes the utilization of proteins, fats, and energy, subsequently impacting animal production, reproduction, and health (2). The molecular responses of livestock to heat stress include: (1) Suppressed feed intake—despite increased energy demands, animals decrease their feed consumption under heat stress, which is mediated by the hypothalamus through upregulation of leptin, adiponectin, and their receptors (3, 4); (2) Mitochondrial damage—heat stress can cause histological and morphological damage to mitochondria, induce fat and protein denaturation, and inhibit cytochrome C release, exacerbating physiological damage (5, 6); (3) Oxidative stress—excessive production of free radicals and reactive oxygen species (ROS) during heat stress damages proteins, lipids, polysaccharides, and DNA, triggering endogenous antioxidant responses and increasing the activity of antioxidant enzymes (2, 7). Therefore, supplementing diets with bioactive compounds exhibiting anti-inflammatory and antioxidant activities could be a promising strategy for mitigating heat stress responses.

*Houttuynia cordata*, commonly known as fish mint, is a perennial herbaceous plant with rhizomes, mainly distributed in Japan, Korea, China, and Southeast Asia (8). It is typically cultivated as leafy greens and rhizome vegetables, especially in humid and shady environments (9). Traditionally, people in these regions use extracts from this plant to treat various ailments. The leaves of *Houttuynia cordata* contain numerous phenolic acids and flavonoids, such as quercetin, chlorogenic acid, methyl linolenate, ferulic acid, and gallic acid, which exhibit anti-inflammatory, antioxidant, antimicrobial, and antiviral properties (10). Yan et al. (11) reported that supplementing fattening pigs with 1 g/kg of HCE improved growth performance, DM and N digestibility, blood white blood cell (WBC) counts, the area of modified live meat, and TBARS values. Hsu et al. (12) found that water extracts of *Houttuynia cordata* leaves could ameliorate glycation and oxidative stress in the hearts and kidneys of diabetic mice. Kang et al. (13) demonstrated that the plant could enhance immune function by modulating splenocyte proliferation and cytokine production in mice. However, there are currently no studies investigating the effects of *Houttuynia cordata* on goat growth performance, antioxidant activity, anti-inflammatory responses, or rumen fermentation. Based on the hypothesis that HCE may elicit comparable effects in goats, this study aimed to examine the effects of supplementing Guizhou black goats with HCE during summer on growth performance, anti-inflammatory activity, and rumen fermentation parameters.

## 2 Materials and methods

### 2.1 Animal ethics statement

**Figure 1:**
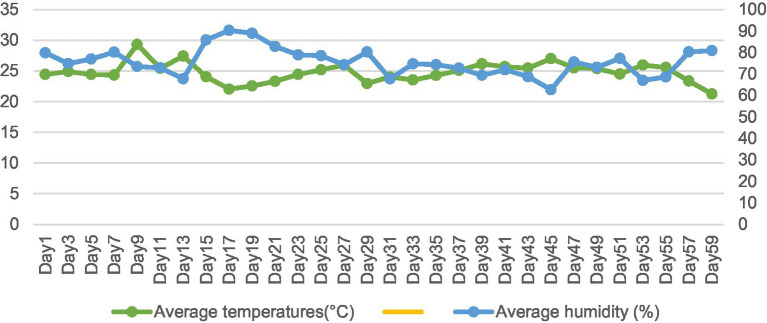
Temperature-humidity index.

Ethical permission for this study was obtained from the Experimental Animal Ethics Committee of Guizhou University (protocol number EAE-GZU-2021-E024). This experiment was carried out in the Maiping Meat Goat Multiplication Farm in Guiyang City, Guizhou Province. The period was from July 1, 2024 to September 14, 2024. The average maximum temperature was 29.31°C, and the average minimum temperature was 21.25°C. The average maximum humidity was 90.28%, and the average minimum humidity was 66.98% (Figure 1).

### 2.2 Experimental animals and design

The extract of *Houttuynia cordata* was purchased from Hunan Nanmo Biological Technology Co., Ltd., containing 98% of neo-volatile compounds as determined by HPLC. A completely randomized single-factor experimental design (CRD) was employed. Twenty-four healthy Guizhou black goats, with similar body weights (16.03 ± 0.79 kg), were randomly divided into three groups, with eight replicates per group and one goat per replicate. The control group (CON) was fed a basal diet. The LC and HC groups received the basal diet supplemented with 500 mg/kg and 1,000 mg/kg of HCE, respectively. The trial lasted for 74 days, including a 14-day pre-feeding period and a 60-day formal feeding period. The basal diet was formulated according to NRC (2007) guidelines and provided twice daily at 8:00 a.m. and 4:00 p.m. The ratio of concentrate to roughage was 60:40, and feed was provided at 3% of body weight. The composition and nutrient levels of the concentrate feed are detailed in Table 1.

**Table 1:** Dietary composition and nutrient composition (dry matter base, %).

Items	Feed composition and nutrient levels
Ingredients	Feed composition and nutritional contents
Corns	64.0
Soybean meal (CP 46%)	20.0
Wheat bran	10.0
CaHPO_4_	0.5
NaCl	0.5
Premix^1^	5.0
Nutrient levels	
GE(kJ/g)	15.33
DM	92.65
OM	91.67
CP	16.44
NDF	18.20
ADF	7.32
Ca	1.13
P	0.95

1 The vitamin-mineral premix was purchased from the Feed Division of Beijing Sanyuan Seed Industry Technology Co., Ltd. (Beijing, China), containing the following per kg: 400 IU VE, 11% Ca, 1,500 mg Mg, 50 mg Cu, 1,500 mg Zn, 1,100 mg Mn, 20 mg P, 5 mg Se and 8 mg Co.

ADF, acidic detergent fibers; CP, crude protein; DM, dry matter; GE, gross energy; NDF, neutral detergent fibers; OM, organic matter.

### 2.3 Chemical composition, growth performance and apparent digestibility

During the final 7 days of the experiment, the diet and feces were collected, dried in a 65°C oven for 72 h, ground, passed through a 1 mm sieve, and stored at 4°C until further analysis. Diet and feces were analyzed for DM, CP, EE, Ca, P, and OM according to the methods of AOAC (14), as well as NDF and ADF (15). Gross energy was calculated using an adiabatic cartridge calorimeter (WGR-WR3, Changsha Benyi Instrument Co., Ltd., Changsha, China). Each sample was assayed in triplicate. The goats were weighed before morning feeding, and the average daily weight gain was calculated for the first and last days of the experiment. Each sample was analyzed in triplicate. Apparent nutrient digestibility was determined using the acid-insoluble ash (AIA) method with the following formula:


$$\begin{array}{l} \text{Apparent nutrient digestibility}\;(\%)=\\ \;\;\;\;\;\;\;\;\;\;\;\;\;\;\;\;\;\;\;\;\;100\%–(\mathrm{AIA}\;\text{in diet}/\mathrm{AIA}\;\text{in feces}\times \text{Nutrient in feces}\\ /\text{Nutrient in diet})\times 100. \end{array}$$

Total Weight Gain=Final Weight–Initial Weight.

$$\begin{array}{l} \text{Average Daily Gain}\;(\mathrm{ADG})=\\ (\text{Final Weight}–\text{Initial Weight})/\text{Number of Experimental Days}. \end{array}$$

$$\begin{array}{l} \mathrm{Dry}\;\text{Matter Intake}\;(\mathrm{DMI})=\\ (\text{Feed Offered}–\text{Feed Refused})/\text{Number of Experimental Days}. \end{array}$$

Feed Conversion Ratio(FCR)=DMI/ADG.

### 2.4 Blood parameters

On day 60 of the experiment, 10 mL of blood was collected via jugular venipuncture from fasted animals using vacuum negative pressure anticoagulant tubes (Guizhou Yu Chili Bioinformation Technology Co., Ltd.). Blood samples were allowed to stand for 30 min and then centrifuged at 4000 × *g* at 4°C for 15 min. The plasma was collected and stored at −80°C for subsequent analysis of plasma parameters. All blood indices were determined using reagent kits from Nanjing Jiancheng Bioengineering Institute. The following reagent kit codes were used for each specific measurement: glutathione peroxidase (GSH-Px, A005-1-2), malondialdehyde (MDA, A003-1-2), total superoxide dismutase (SOD, A001-3-2), total antioxidant capacity (T-AOC, A015-2-1), catalase (CAT, A007-1-1), total protein (TP, A045-2-2), albumin (Alb, A028-2-1), blood urea nitrogen (BUN, C013-2-1), glucose (Glu, F006-1-1), immunoglobulin A (IgA, H018-1-2), immunoglobulin M (IgM, H019-1-2), and immunoglobulin G (IgG, H016-1-2), Interleukin-6 (IL-6, H007-1-1), interleukin-8 (IL-8, H008-1-1), interleukin-10 (IL-10, H009-1-2), interleukin-1β (IL-1β, H002-1-2), and tumor necrosis factor-*α* (TNF-α, H052-1-2).

### 2.5 Rumen parameters

On the last day of the experiment, 50 mL of rumen fluid was collected before morning feeding using a vacuum pump connected to a stomach tube. To avoid contamination, the first 50 mL of rumen fluid was discarded. The pH value was immediately measured using a portable pH meter (Mettler Toledo). The rumen fluid was then filtered through four layers of cheesecloth. A portion of the filtrate (5 mL) was mixed with 1 mL of 15% metaphosphoric acid and stored at −20°C for the analysis of rumen volatile fatty acids (VFAs) and ammonia nitrogen (NH_3_-N). VFAs were analyzed according to the method of Suong et al. (16). The concentration of VFAs in the filtrate was determined by gas chromatography (Agilent 6,890 GC, Agilent Technologies, Santa Clara, CA) using a silica capillary column (30 m × 250 μm × 0.25 μm). The initial oven temperature was set to 40°C and held for 2 min, then increased to 100°C at a rate of 3.5°C/min, followed by an increase to 249.8°C at a rate of 10°C/min. The total run time was 30 min. The vaporization chamber temperature was 250°C, and helium (99.99%) was used as the carrier gas at a pressure of 31.391 psi. The carrier gas flow rate was 3.0 mL/min, and the solvent delay time was 3 min. MS conditions were set to single ion monitoring scan mode, with ion energy and ionization energy at 70 eV. The NH_3_-N concentration in rumen fluid was analyzed using a colorimetric method (17).

### 2.6 Statistical analysis

Data were analyzed using SPSS statistical software (Windows version 27.0; IBM SPSS, Chicago, Illinois, USA). One-way analysis of variance (ANOVA) was performed to assess differences among groups. Multiple comparisons were conducted using Tukey’s Honestly Significant Difference (HSD) test to determine statistical significance. Data were presented as means ± standard error of the mean (SEM), with a significance threshold set at *p* < 0.05, *p* < 0.1 indicated a trend.

## 3 Results

### 3.1 Effect of HCE on the growth performance of Guizhou black goats

The effects of HCE on the growth performance of Guizhou black goats are presented in Table 2. The ADG in the HC group was significantly higher (*p* < 0.05) than that in the CON group. With the supplementation of HCE, DMI showed an increasing trend (*p* = 0.057). However, the HCE did not have a significant (*p* > 0.05) effect on final weight and FCR.

**Table 2:** Effect of HCE on the growth performance of Guizhou black goat.

Items	CON	LC	HC	SEM	*p*-value
Initial weight (kg)	16.10	16.08	15.90	0.16	0.875
Final weight (kg)	24.23	24.90	25.13	0.23	0.253
Total weight gain (kg)	8.13b	8.83ab	9.23a	0.16	0.009
ADG (g)	135.42b	147.08ab	153.75a	2.63	0.009
DMI (g)	1025.00	1095.00	1118.75	16.98	0.057
FCR	7.59	7.49	7.29	0.12	0.631

ADG, average daily weight gain; DMI, dry matter feed intake; FCR, feed conversion ratio. Lowercase letters (e.g., a, b) in table notes indicate statistically significant differences (*p* < 0.05).

### 3.2 Effect of HCE on the apparent digestibility in Guizhou black goats

The impact of HCE on the apparent digestibility in Guizhou black goats is presented in Table 3. The digestibility of DM in both the LC and HC groups was significantly higher (*p* < 0.05) than in the CON group. Additionally, the digestibility of CP, GE, and NDF in the HC group was significantly higher (*p* < 0.05) than in the CON group. There were no significant differences (*p* > 0.05) among groups in the digestibility of OM and ADF.

**Table 3:** Effect of HCE on the apparent digestibility of Guizhou black goats (%).

Items	CON	LC	HC	SEM	*p*-value
DMD	66.33b	69.78a	70.02a	0.53	0.002
OMD	70.48	72.25	72.43	0.41	0.100
CPD	75.45b	79.12ab	79.46a	0.71	0.027
GED	67.13b	70.00ab	70.88a	0.62	0.003
NDFD	42.71b	44.43ab	46.27a	0.58	0.014
ADFD	33.13	34.32	33.59	0.37	0.417

ADFD, acidic detergent fibers digestibility; CPD, crude protein digestibility; DMD, dry matter digestibility; GED, gross energy digestibility; NDFD, neutral detergent fibers digestibility; OMD, organic matter digestibility. Lowercase letters (e.g., a, b) in table notes indicate statistically significant differences (*p* < 0.05).

### 3.3 Effect of HCE on the antioxidant parameters in Guizhou black goats

The impact of HCE on antioxidant parameters in Guizhou black goats is detailed in Table 4. GSH-Px levels in both the LC and HC groups were significantly higher (*p* < 0.05) than in the CON group. With the supplementation of HCE, SOD showed an increasing trend (*p* = 0.067). T-AOC in the HC group was also significantly higher (*p* < 0.05) than in the CON group. MDA levels in the HC group were significantly lower (*p* < 0.05) than in both the LC and CON groups (*p* < 0.05), and MDA levels in the LC group were also significantly lowe*r* (*p* < 0.05) than in the CON group.

**Table 4:** Effect of HCE on the antioxidant parameters in Guizhou black goat.

Items	CON	LC	HC	SEM	*p*-value
GSH-Px (U/mL)	507.81b	628.78a	680.75a	18.89	<0.001
SOD (U/mL)	109.52	118.75	119.40	1.97	0.067
CAT (U/mL)	1.07	1.06	1.12	0.03	0.767
T-AOC (mmol/mL)	0.16b	0.18ab	0.20a	0.007	0.036
MDA (mmol/mL)	2.15a	1.78b	1.40c	0.07	<0.001

Lowercase letters (e.g., a, b, c) in table notes indicate statistically significant differences (*p* < 0.05).

### 3.4 Effect of HCE on the plasma biochemical and immune parameters in Guizhou black goats

The effects of HCE on plasma biochemical and immune parameters are shown in Table 5. The TP content in the HC group was significantly higher (*p* < 0.05) than in the CON group. IgA levels in both the LC and HC groups were significantly higher (*p* < 0.05) than in the CON group. Other biochemical and immune parameters showed no significant differences (*p* > 0.05) among the groups.

**Table 5:** Effect of HCE on the plasma biochemical and immune parameters of Guizhou black goat.

Items	CON	LC	HC	SEM	*p*-value
TP (g/L)	44.26b	46.89ab	48.21a	0.56	0.007
Alb (g/L)	23.92	23.76	24.14	0.55	0.965
BUN (mmol/L)	3.95	3.92	4.22	0.08	0.289
Glu (mmol/L)	3.93	4.05	4.24	0.09	0.367
IgA (g/mL)	45.78b	52.86a	57.63a	0.45	<0.001
IgM (g/mL)	639.57	663.45	675.94	13.47	0.555
IgG (g/mL)	653.31	695.50	699.60	10.24	0.122

Lowercase letters (e.g., a, b) in table notes indicate statistically significant differences (*p* < 0.05).

### 3.5 Effect of HCE on the inflammatory markers in Guizhou black goats

The effects of HCE on inflammation in Guizhou black goats are shown in Table 6. The levels of IL-6 and IL-8 in the HC group were significantly lower (*p* < 0.05) than in the LC and CON groups. Interestingly, the IL-10 level in the HC group was significantly higher (*p* < 0.05) than in the LC and CON groups, and the IL-10 level in the LC group was significantly higher (*p* < 0.05) than in the CON group. The TNF-*α* level in the HC group was significantly lower (*p* < 0.05) than in the CON group.

**Table 6:** Effect of HCE on the inflammation in Guizhou black goat (pg/mL).

Items	CON	LC	HC	SEM	*p*-value
IL-6	48.21a	45.62a	39.39b	0.89	<0.001
IL-8	41.47a	40.85a	37.32b	0.50	<0.001
IL-1	16.27c	20.58b	23.19a	0.67	<0.001
IL-1β	27.71	27.05	26.16	0.37	0.249
TNF-*α*	84.94a	79.89ab	78.35b	1.02	0.014

Lowercase letters (e.g., a, b) in table notes indicate statistically significant differences (*p* < 0.05).

### 3.6 Effect of HCE on the rumen pH, NH_3_-N, and VFAs in Guizhou black goats

The effects of HCE on rumen pH, NH_3_-N, and VFAs in Guizhou black goats are presented in Table 7. The HCE had no significant (*p* > 0.05) effect on rumen pH and VFAs. However, with the supplementation of HCE, NH_3_-N showed an increasing trend (*p* = 0.078).

**Table 7:** Effects of HCE on the rumen pH, NH_3_-N and VFA of Guizhou black goat.

Items	CON	LC	HC	SEM	*p*-value
pH	6.17	6.18	6.32	0.04	0.204
NH_3_-N(mg/dL)	9.97	10.82	11.48	0.28	0.078
VFA					
Total VFA(mmol/L)	59.90	54.18	62.05	1.35	0.454
Acetic acid(mmol/L)	39.84	35.29	40.42	0.67	0.291
Propionic acid(mmol/L)	11.42	10.83	13.86	0.25	0.406
Butyric acid(mmol/L)	8.64	8.06	7.77	1.53	0.297
Acetic acid/ propionic acid	3.61	3.38	3.14	0.18	0.553

## 4 Discussion

While this study sheds light on the effects of HCE on growth performance, blood parameters, and rumen fermentation in goats, interpretations of the findings warrant caution due to the limited sample size (*n* = 8). The modest sample size may have resulted in insufficient statistical power to detect subtle yet potentially significant biological effects of HCE. Consequently, the overall impact of HCE on certain metabolic pathways might have been underestimated. For instance, while no significant differences were observed in rumen parameters, this does not necessarily indicate a complete lack of effect; rather, the limited statistical power may have precluded the detection of subtle modulating effects. Future studies employing larger cohorts and a more nuanced experimental design are warranted to comprehensively evaluate the robustness of these observations and fully elucidate the complex interplay between HCE, the rumen microbiome, and host physiology.

### 4.1 Effect of HCE on the growth performance of Guizhou black goats

Animal growth performance is directly linked to economic benefits. In the context of the ban on antibiotics, herbal medicines are widely recommended to livestock producers as alternatives to antibiotic growth promoters (18). Redoy et al. (19) reported that supplementing goats with herbal supplements increased live weight gain and feed conversion ratio by 18 and 13%, respectively. Hashemzadeh et al. (20) found that supplementing heat-stressed lambs with a herbal mixture improved DMI, ADG, FCR, and body weight. Wang et al. (21) reported that supplementing cattle with a mixture of traditional Chinese herbs resulted in faster growth. In agreement with these previous findings, our study showed that the HC group had significantly higher total weight gain and ADG compared to the control group, with the supplementation of HCE, DMI showed an increasing trend, suggesting that HCE promoted growth performance in goats. This could be because herbal additives improved the flavor and palatability of feed, thereby increasing overall feed intake and growth performance (22). Furthermore, it’s possible that HCE enhanced the immune, antioxidant, and anti-inflammatory responses in goats, which in turn promoted their growth performance (23).

### 4.2 Effect of HCE on the apparent digestibility in Guizhou black goats

The digestibility of nutrients is a key parameter for evaluating feed quality. The results of this study showed that supplementing with HCE improved the apparent digestibility of DM, GE, and NDF. These findings are consistent with those of Kholif and Olafadehan (24), who found that supplementation with lemongrass and rosemary increased the digestibility of OM, NDF, ADF, and hemicellulose in goats. Du et al. (25) reported that diets supplemented with traditional Chinese herbal medicines enhanced the digestibility of DM, OM, CP, and NDF in lambs. Additionally, Dwatmadji et al. (26) observed that added herbal and humic acid supplements increased dry matter intake and improved digestibility indicators in goats. These studies suggest that HCE can promote the digestion of nutrients in goats. This may be due to its potential to enhance the activity of ruminal digestive enzymes, thereby increasing apparent digestibility (27). Furthermore, HCE may increase the abundance of beneficial rumen microbes (28).

### 4.3 Effect of HCE on the antioxidant parameters in Guizhou black goats

Under high-temperature conditions, animals experience oxidative stress. Oxidative stress is primarily viewed as an imbalance between the production of reactive oxygen species (ROS) and the body’s mechanisms for their elimination (29). Enzymes such as cytochrome P450, GSH-Px, SOD, and CAT play crucial roles in scavenging free radicals. Numerous studies have demonstrated the potent antioxidant properties of HCE both *in vivo* and *in vitro* (30). The results of this study indicated that supplementing with HCE increased GSH-Px levels, and there was a trend toward increased SOD levels. Additionally, high doses of the extract were effective in enhancing T-AOC while reducing MDA levels. These findings are consistent with those of Hsu et al. (12), who observed that supplementing with 2% HCE decreased ROS levels in the heart and kidneys of mice. Another study reported by Kang et al. (13) found that oral administration of 500 mg/kg/day or 1,000 mg/kg/day of HCE significantly improved the levels of GSH-Px, SOD, CAT, and MDA in the kidneys of rats subjected to oxidative stress and nephrotoxicity induced by daphnetin. Currently, no reports regarding the application of *Houttuynia cordata* in ruminants have been found. However, a meta-analysis reported that supplementation with flavonoids (primarily composed of anthocyanins and daidzein) resulted in a reduction in serum MDA concentrations and an increase in SOD, GSH-Px, and T-AOC in ruminants (31). Similar results were obtained in other studies, where dietary supplementation with anthocyanins was shown to enhance plasma antioxidant capacity in goats (32). Furthermore, it has been demonstrated that flavonoids can induce the activation of the transcription factor Nrf2, which is capable of activating the activity of several antioxidant enzymes (33). This antioxidant effect may be attributed to the flavonoid and polyphenol components in *Houttuynia cordata*, which enhanced antioxidant capacity by scavenging ROS, increasing antioxidant enzyme activities, and chelating metal ions (34, 35). Additionally, *Houttuynia cordata* polysaccharides have been found to improve antioxidant capacity by regulating antioxidant enzymes, scavenging ROS, and antagonizing NO via the Nrf2/ARE pathway (36).

### 4.4 Effect of HCE on the plasma biochemical and immune parameters in Guizhou black goats

In thermally stressful environments, animals experience oxidative stress, which can compromise their immune function. TP, ALB, and BUN are key indicators of CP digestion and absorption in goats, while Glu reflects energy metabolism (37). Plasma IgG, IgM, and IgA are crucial products of the immune response against antigens (38). Our study found that HCE elevated plasma TP and IgA levels, suggesting that it promoted protein turnover and enhanced immune function in goats. The immunomodulatory effects of HCE appear to be primarily mediated through its antioxidant and anti-inflammatory properties. First, due to its richness in antioxidant components like vitamin C, polyphenols, flavonoids, and polysaccharides, HCE exhibits strong antioxidant and lipid peroxidation inhibitory activity, effectively scavenging free radicals and preventing oxidative stress (39). Second, *Houttuynia cordata* polysaccharides can modulate innate immunity by enhancing the function of activated macrophages and the phagocytic activity of leukocytes, playing a therapeutic role in anti-inflammatory and anti-tumor processes (40). Furthermore, the purified pectic polysaccharide HCP-2, isolated from *Houttuynia cordata* water extract, can be used as an immunostimulant, significantly increasing serum concentrations of IL-1β, TNF-*α*, MIP-1α, and MIP-1β, thereby promoting immune function (41).

### 4.5 Effect of HCE on the inflammatory markers in Guizhou black goats

Heat stress significantly impacts inflammation in animals. Volatile compounds, essential oils, polysaccharides, and bioactive molecules extracted from *Houttuynia cordata*, such as sodium houttuyfonate and 2-undecanone, have demonstrated anti-inflammatory properties against various inflammatory conditions (30). Our study revealed that the HC group experienced reduced levels of IL-6, IL-8, and TNF-*α*, along with elevated IL-10 levels. This suggested that HCE enhanced the anti-inflammatory response in goats subjected to heat stress. This finding was consistent with the results obtained by Urkmez et al. (42), who investigated the effects of supplementing heat-stressed calves with 50 and 100 mg/kg grape seed extract on antioxidant activity and inflammatory responses. Their study revealed a reduction in plasma MDA and TNF-*α* levels in the heat-stressed calves. Similarly, Zhang et al. (43) explored the antioxidant effects of a polysaccharide-rich *Morinda citrifolia* fruit extract in goats, finding that dietary inclusion of 0.4% extract increased serum TNF-*α* and IL-6 concentrations. These beneficial effects are generally attributed to plant bioactive compounds, which are known to induce Nrf2 nuclear translocation, thereby improving the Nrf2 signaling pathway. This, in turn, leads to an increased expression of downstream target genes (such as GSH-Px and SOD) and the inhibition of NF-κB activity in the liver, thus reducing the concentrations of pro-inflammatory cytokines (e.g., TNF-α, IL-6, and IFN-*γ*) and exerting anti-inflammatory effects (44).

### 4.6 Effects of HCE on the rumen pH, NH_3_-N and VFA of Guizhou black goat

Rumen pH is a crucial indicator of nutritional metabolism and digestive environment homeostasis, with a normal range of 5.0–7.5 (45). Rumen microbes break down nitrogenous substances, creating intermediate products. NH_3_-N originates from the degradation of feed protein and is utilized for the synthesis of microbial protein. Its optimal concentration ranges from 2.37 to 27.3 mg/dL, serving as the most important nitrogen source for ruminants (46). VFAs are the primary products of rumen fermentation and are positively correlated with substrate digestibility, accounting for approximately 40 to 70% of digestible energy intake. They represent a major energy source for ruminants (47). In this study, all rumen parameters fell within the normal range across all treatments, but the differences were not statistically significant. This suggests that HCE may not significantly affect the rumen environment. However, NH₃-N showed a trend of increase, indicating a promoting effect on microbial protein synthesis. This also explained the significant increase in CP digestibility observed in this study. The differences observed may have been due to the HC group primarily exerting its effects in the small intestine.

## 5 Conclusion

During the summer, the inclusion of 1,000 mg/kg of HCE in the diet of Guizhou black goats was found to improve the apparent digestibility of nutrients and bolster their antioxidant, immune, and anti-inflammatory functions, leading to enhanced animal growth. The results of this study suggest that future investigations should validate HCE’s capabilities in diverse seasons, with different goat breeds, and at elevated supplementation levels.

## Data Availability

The raw data supporting the conclusions of this article will be made available by the authors, without undue reservation.

## Footnotes

^1^ https://mirror-earth.com
